# Pre-transplant measurable residual disease by flow cytometry is an independent prognostic factor in pediatric acute myeloid leukemia undergoing allogeneic hematopoietic stem cell transplantation

**DOI:** 10.3389/fonc.2026.1864716

**Published:** 2026-07-17

**Authors:** Shaoyang Deng, Shan He, Na Song, Zhijun Huang, Benshan Zhang

**Affiliations:** Department of Hematology, The Affiliated Children’s Hospital of Xiangya School of Medicine, Central South University (Hunan Children’s Hospital), Changsha, Hunan, China

**Keywords:** pediatric acute myeloid leukemia, allogeneic hematopoietic stem cell transplantation, measurable residual disease, flow cytometry, prognosis, high‑risk fusion genes

## Abstract

**Background:**

Allogeneic hematopoietic stem cell transplantation (allo-HSCT) is a critical treatment for pediatric acute myeloid leukemia (AML); however, relapse after transplantation remains a major challenge. This study aimed to evaluate the prognostic value of pre-transplant measurable residual disease (MRD) detected by multiparameter flow cytometry (MFC) and high-risk (HR) fusion genes on survival after transplantation.

**Methods:**

This single-center retrospective study included 80 newly diagnosed pediatric AML patients who underwent allo-HSCT during their first complete remission between October 2019 and October 2025. All patients were treated according to the C-HUANAN-AML 15 protocol prior to transplantation, with risk stratification and treatment decisions based on European LeukemiaNet criteria and serial MFC-MRD assessments. Cox regression models were employed for statistical analysis.

**Results:**

With a median follow-up of 34.5 months, the 3-year disease-free survival (DFS) and overall survival (OS) rates were 85.5% ± 4.2% and 86.8% ± 4.1%, respectively. Univariate analysis identified several risk factors for inferior survival, but multivariate analysis confirmed pre-transplant MFC-MRD positivity as an independent adverse prognostic factor for both 3-year DFS and OS (DFS: HR = 14.304, 95%CI: 1.892–108.155, *P* = 0.010; OS: HR = 15.847, 95%CI: 2.036–123.328, *P* = 0.008). Among relapsed patients, the majority harbored HR fusion genes. Given the limited number of cases with specific HR fusion genes (e.g., *NUP98* rearrangements, n = 4; *FUS*::*ERG*, n = 2), these subgroup findings are exploratory and require validation in larger cohorts.

**Conclusion:**

Pre-transplant MFC-MRD positivity is an independent adverse prognostic marker for survival in pediatric AML patients following allo-HSCT. Patients harboring HR fusion genes such as *NUP98* rearrangements or *FUS*::*ERG* fusions may remain at risk of relapse even after successful transplantation, although this observation is based on a limited number of cases. These findings emphasize the importance of achieving deep remission to reduce tumor burden before transplantation and provide a rationale for intensified post-transplant management strategies, including maintenance therapy, for this HR population.

## Introduction

Acute myeloid leukemia (AML) is one of the most common malignancies in children (1, 2). Although the prognosis of pediatric AML has significantly improved due to optimized chemotherapy, precision risk stratification, the incorporation of targeted therapies, and advances in supportive care, approximately 30%–40% of patients still experience relapse (3–6). Allogeneic hematopoietic stem cell transplantation (allo-HSCT) provides a critical curative opportunity for children with high-risk and selected intermediate-risk AML, which exerts its effect primarily through the graft-versus-leukemia (GVL) mechanism to eliminate residual leukemic cells. However, even after transplantation, a subset of patients remains at risk of relapse, which represents the leading cause of mortality in these children (7, 8). Therefore, the precise identification of key prognostic factors influencing transplant outcomes is essential for optimizing the timing of transplantation, guiding appropriate interventions, and improving post-transplant management.

In recent years, multiple studies have confirmed that measurable residual disease (MRD) positivity at various stages of AML treatment, particularly after induction chemotherapy and before transplantation, is closely associated with an increased risk of relapse (9–12). Consequently, MRD has become a powerful prognostic indicator in AML. The most widely used methods for MRD monitoring in clinical practice are currently multiparameter flow cytometry-based MRD (MFC-MRD) and molecular MRD (Mol-MRD) assessed by reverse transcription polymerase chain reaction (RT-PCR) (13). Although Mol-MRD achieves a high sensitivity of up to 10^−5^, it is applicable to only approximately 50% of patients who harbor suitable RNA-based fusion targets (14). In contrast, MFC-MRD, with a relatively lower sensitivity ranging from 10^−3^ to 10^−4^, can be applied to nearly all AML patients, making it the most broadly utilized approach for MRD detection. In adult AML, pre-transplant MFC-MRD positivity is widely recognized as a marker of poor prognosis (15). However, within the pediatric AML population, the prognostic impact of MFC-MRD positivity appears to vary across different treatment phases (16–18). Furthermore, most existing studies have focused either on outcomes during chemotherapy or on the peri-transplant period, while dedicated investigations that systematically evaluate the influence of serially monitored MFC-MRD positivity—across consecutive chemotherapy stages and at the time of transplantation—on outcomes in children with AML remain relatively limited. Additionally, beyond MFC-MRD status, factors such as disease genetic characteristics and transplant modalities may interact to collectively influence transplantation outcomes.

Acute promyelocytic leukemia (APL) was excluded from this study because it is treated with targeted therapy (all-trans retinoic acid and arsenic trioxide) rather than conventional AML chemotherapy, and its underlying biology, transplant indications, and post-transplant outcomes differ substantially from other pediatric AML subtypes.

This study aimed to retrospectively analyze the clinical data of pediatric patients with newly diagnosed AML who underwent allo-HSCT at a large pediatric hematology center. We focused on evaluating the independent and combined prognostic value of pre-transplantation MFC-MRD status and HR fusion genes. The research seeks to identify key determinants of post-transplant survival to inform risk-adapted transplantation strategies and post-transplant management, including the potential use of maintenance therapy for HR patients.

## Methods

### Patients

This retrospective study analyzed 80 consecutive pediatric patients with newly diagnosed AML who underwent allo-HSCT at Hunan Children’s Hospital from October 2019 to October 2025. At initial diagnosis, all patients completed a comprehensive diagnostic workup comprising bone marrow morphology, flow cytometric immunophenotyping, cytogenetic analysis, and molecular profiling. Inclusion criteria were: (1) newly diagnosed AML according to the 2016 WHO classification; (2) age ≤ 18 years at diagnosis; and (3) completion of protocol-based chemotherapy followed by allo-HSCT. Exclusion criteria included: (1) relapsed AML; (2) non-protocol-based chemotherapy; (3) failure to undergo allo-HSCT; (4) Acute promyelocytic leukemia; (5) lost to follow-up. The study protocol received approval from the Institutional Review Board of Hunan Children’s Hospital (HCHLL-2026-15). Written informed consent for all treatment-related procedures was obtained from parents or legal guardians in accordance with the principles of the Declaration of Helsinki.

### Chemotherapy regimen

Chemotherapy was administered according to the C-HUANAN-AML 15 protocol, which was based on the framework of the Medical Research Council (MRC) AML-15 regimen. Details of the treatment regimen are provided in **Supplementary Figure 1**. Bone marrow morphology and MFC-MRD were assessed on days 28–35 of each treatment cycle, once the neutrophil count recovered to ≥ 0.5 × 10^9^/L. If a patient had an indication for allo-HSCT, transplantation was recommended after the first cycle of consolidation chemotherapy. Patients with *FLT3-ITD* mutations received additional targeted therapy with sorafenib, which was continued as maintenance therapy for two years post-transplantation.

### Risk stratification

Risk stratification integrated both genetic abnormalities and early morphological treatment response. Genetic risk was classified according to the European LeukemiaNet (ELN) 2017 criteria for patients treated between 2019–2021 (19), and the ELN 2022 criteria for those treated from 2022 onward (15). Patients with favorable genetic features who achieved complete remission (CR) after the first induction course were assigned to the low-risk (LR) group. The high-risk (HR) group included patients with adverse-risk genetic abnormalities, as well as those who had ≥ 15% bone marrow blasts after the first induction or failed to achieve CR after the second induction, irrespective of their genetic profile. The intermediate-risk (IR) group comprised the remaining patients after excluding those meeting the LR or HR criteria.

### Indications for allo-HSCT

Indications for allo-HSCT comprised all IR and HR group patients, as well as LR group patients who exhibited MFC-MRD ≥ 1% after the first induction cycle or MFC-MRD ≥ 0.1% after the second induction cycle.

### Conditioning regimen

All patients received a myeloablative conditioning regimen based on busulfan, cyclophosphamide, fludarabine, and rabbit anti-human thymocyte immunoglobulin (ATG). For HLA-haploidentical HSCT, cyclophosphamide and fludarabine doses were adjusted. GVHD prophylaxis consisted of cyclosporine, mycophenolate mofetil, and methotrexate, with post-transplant cyclophosphamide added for HLA-haploidentical recipients. A detailed description of the conditioning regimen and GVHD prophylaxis, including drug dosages and schedules, is provided in **Supplementary Table 1**.

### Molecular analyses for gene fusions and *FLT3-ITD*

Gene fusions were detected using targeted RNA sequencing (Archer FusionPlex Pan-Heme Kit, ArcherDX, Boulder, CO, USA) with an average sequencing depth of 5 million reads. *FLT3-ITD* mutations were identified by PCR amplification of the *FLT3* gene (exons 14–15) followed by capillary electrophoresis fragment analysis, with an *ITD* ratio ≥ 0.05 considered positive (20).

### Monitoring of MFC-MRD

Bone marrow samples were collected from all patients at 28–35 days after each chemotherapy cycle (when the neutrophil count recovered to ≥ 0.5 × 10^9^/L) for MFC-MRD evaluation. An 8-color MFC panel containing antibodies against the specified markers was used for assessment. Analyses were performed on a FACSCanto flow cytometer (Becton Dickinson, Franklin Lakes, NJ, USA). The antibody panel consisted of the following two combinations: (a) CD38-FITC, CD117-PE, CD34-PerCP-Cy5.5, CD33-PE-Cy7, CD13-APC, HLA-DR-APC-Cy7, CD11b-V450, and CD45-V500; (b) CD15-FITC, CD34-PE, CD56-PerCP-Cy5.5, CD33-PE-Cy7, CD7-APC, CD14-APC-Cy7, CD19-V450, and CD45-V500. MFC-MRD employed two complementary approaches: (a) leukemia-associated immunophenotype (LAIP) method; (b) differentiation/maturation (DFN) abnormality method. For MFC-MRD detection, a minimum of 500, 000 events were collected and analyzed. A cluster containing at least 50 events was considered distinguishable leukemic cells from normal cells.

### Definitions and statistical analysis

CR was defined as bone marrow blasts < 5%, with the absence of auer rods, no evidence of extramedullary disease, and recovery of peripheral blood counts (3). Relapse was defined as the recurrence of bone marrow blasts ≥ 5%, reappearance of blasts in peripheral blood, or the development of extramedullary disease after an initial morphologic CR (3). Overall survival (OS) was calculated from the date of initial diagnosis to death from any cause or the last follow-up. Disease-free survival (DFS) was calculated from the date of initial diagnosis to the first event, defined as relapse, death from any cause, or the last follow-up. For both OS and DFS, patients who were alive and event-free at the data cutoff date (October 31, 2025) were censored at their last known follow-up date. MFC-MRD positivity was defined as the presence of ≥ 0.1% leukemia-associated immunophenotype cells.

Statistical analyses were performed using SPSS software (version 25.0). Continuous variables that followed a normal distribution are presented as mean ± standard deviation (SD), while non-normally distributed variables are expressed as median (range). Categorical variables are summarized as frequencies and percentages. Survival curves were generated using the Kaplan-Meier method, and comparisons between groups were made with the log-rank test. Variables that showed a significant association (*P* < 0.05) in univariate analysis were subsequently entered into a Cox proportional hazards regression model for multivariate analysis. Given the limited number of events (particularly only 6 pre-transplant MRD positive cases), the multivariate analysis should be considered exploratory, and the results require validation in larger prospective cohorts. A two-sided P-value < 0.05 was considered statistically significant. Figures were created using GraphPad Prism software (version 10).

## Results

### General clinical characteristics

A total of 80 pediatric AML patients were enrolled in this study, all of whom underwent allo-HSCT during their first CR. The cohort consisted of 42 males (52.5%) and 38 females (47.5%), with a median age of 8.1 years (range: 1–17). Regarding other baseline characteristics, 28 patients (35.0%) were aged ≥ 10 years, 13 patients (16.3%) presented with a white blood cell (WBC) count ≥ 50×10^9^/L, 28 patients (35.0%) were classified as HR by ELN criteria, 54 patients (67.5%) received haplo-HSCT, and 26 (32.5%) received HLA-matched HSCT. For detailed distributions, please refer to **Table 1**.

**Table 1:** Baseline characteristics of the patients stratified by 3-year DFS and OS status.

Baseline characteristics	3-yr DFS rate (± %)	χ^2^	P	3-yr OS rate (± %)	χ2	P
Sex (n)		4.305	0.038		3.205	0.073
Male (42)	94.0 ± 4.1			93.8 ± 4.3		
Female (38)	77.0 ± 7.2			79.7 ± 6.9		
Age (n)		0.257	0.612		0.590	0.442
≥ 10 years (28)	83.0 ± 7.8			82.8 ± 7.9		
< 10 years (52)	86.9 ± 5.0			88.9 ± 4.7		
WBC at diagnosis (n)		0.099	0.753		0.230	0.631
≥ 50 × 109/L (13)	82.5 ± 11.3			91.7 ± 8.0		
< 50 × 109/L (67)	86.2 ± 4.6			85.8 ± 4.7		
Risk group* (n)		3.271	0.071		4.860	0.027
HR group (28)	76.0 ± 8.6			74.9 ± 9.0		
Non-HR group (52)	90.8 ± 4.4			93.2 ± 3.8		
Karyotype (n)		0.329	0.566		0.623	0.430
Normal	80.8 ± 10.0			80.2 ± 10.3		
Abnormal	86.8 ± 4.7			88.6 ± 4.4		
FLT3-ITD mutation (n)		1.767	0.184		1.518	0.218
Negative (69)	83.2 ± 4.9			84.7 ± 4.7		
Positive (11)	100			100		
KMT2A-r excluded MLLT3::KMT2A (n)		0.965	0.326		0.697	0.404
Negative (63)	83.1 ± 5.2			84.8 ± 5.0		
Positive (17)	93.8 ± 6.1			93.8 ± 6.1		
CBFβ::MYH11 (n)		0.762	0.383		0.638	0.424
Negative (73)	84.5 ± 4.5			85.9 ± 4.4		
Positive (7)	100			100		
RUNX1::RUNX1T1 (n)		0.359	0.549		0.190	0.663
Negative (60)	83.7 ± 5.3			85.4 ± 5.2		
Positive (20)	89.7 ± 6.9			89.7 ± 6.9		
HR fusion genes* (n)		4.891	0.027		6.558	0.010
Positive (24)	72.7 ± 9.6			71.9 ± 9.8		
Negative (56)	91.4 ± 4.1			93.6 ± 3.6		
MFC-MRD after cycle 1 induction		0.721	0.396		0.237	0.626
Positive (30)	80.6 ± 7.8			83.9 ± 7.4		
Negative (50)	88.5 ± 4.8			88.4 ± 4.9		
MFC-MRD after cycle 2 induction (n)		4.636	0.031		5.400	0.020
Positive (9)	62.5 ± 17.1			62.5 ± 17.1		
Negative (71)	88.5± 4.1			90.0 ± 3.9		
Pre-transplant MFC-MRD (n)		30.946	< 0.001		33.320	< 0.001
Positive (6)	20.0 ± 17.9			20.0 ± 17.9		
Negative (74)	90.5 ± 3.7			92.0 ± 3.5		
Haplo-HSCT (n)		0.402	0.526		0.011	0.917
Yes (26)	87.8 ± 4.7			87.6 ± 4.8		
No (54)	80.8 ± 8.7			85.6 ± 7.7		
Acute GVHD (n)		1.114	0.291		0.713	0.399
Yes (26)	91.8 ± 5.5			91.8 ± 5.5		
No (54)	82.0 ± 5.8			84.0 ± 5.6		
Chronic GVHD (n)		2.425	0.119		2.216	0.137
Yes (13)	100			100		
No (67)	82.3 ± 5.1			83.8 ± 5.0		

yr, year; DFS, disease-free survival; OS, overall survival; HR, high risk; n, number; MFC, multiparametric flow cytometry; MRD, measurable residual disease; Haplo-HSCT, haploidentical hematopoietic stem cell transplantation; GVHD, graft versus host disease; ^*^European leukemia network 2022 risk classification.

### Cytogenetic and molecular characteristics

Chromosomal karyotyping identified a normal karyotype in 18 (22.5%) cases and an abnormal karyotype in 62 (77.5%) cases. Gene fusions were detected in 59 (73.8%) patients, encompassing 18 distinct fusion gene types. Gene mutations were identified in 66 (82.5%) patients, involving 18 different mutated genes. For detailed distributions of specific karyotypic abnormalities, fusion genes, and gene mutations, please refer to **Supplementary Table S2**.

### Results of CR and MFC-MRD at various treatment stages

CR was achieved in 75 patients (93.7%) after the first induction chemotherapy and in all 80 patients (100%) after the second induction. MFC-MRD status was assessed at serial time points. After the first induction, 30 patients (37.5%) were MFC-MRD positive and 50 (62.5%) were negative. Following the second induction, 9 patients (11.3%) were positive and 71 (88.7%) were negative. Immediately prior to transplantation, 6 patients (7.5%) were MFC-MRD positive, while 74 (92.5%) were negative. At one month post-transplantation, only 1 patient (1.3%) remained MFC-MRD positive, and 79 (98.7%) were negative.

### Survival outcomes

**Figure 1:**
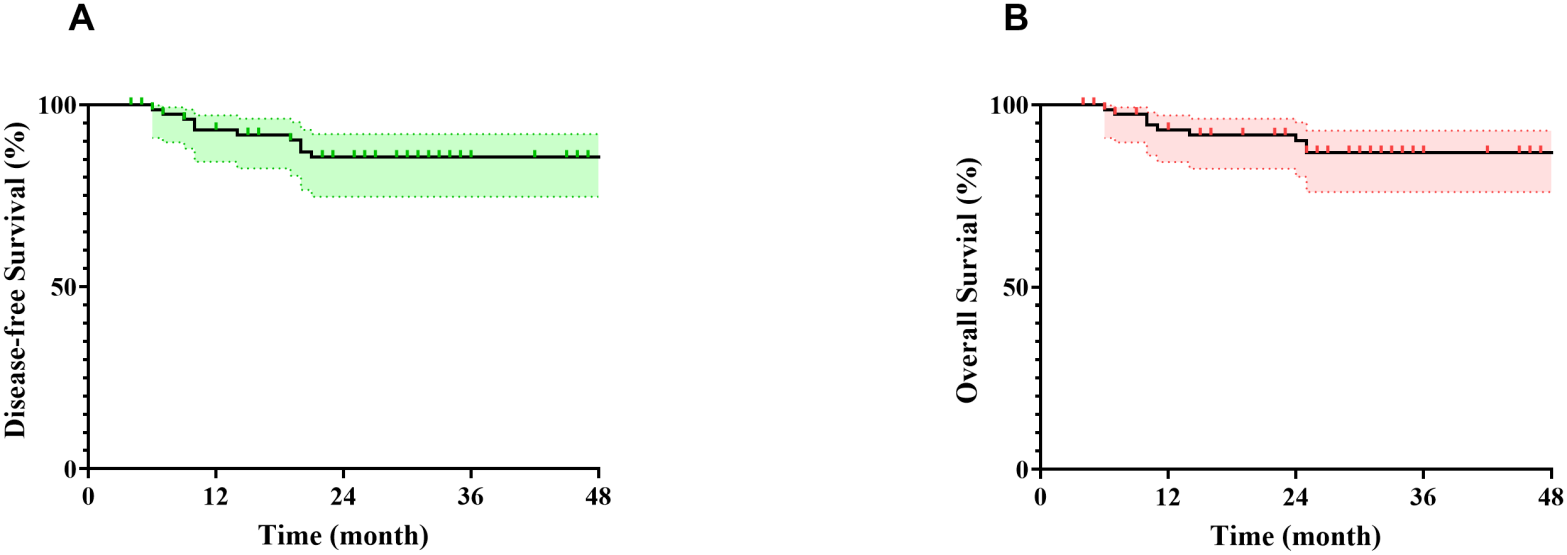
Survival outcomes of 80 pediatric patients who underwent allogeneic hematopoietic stem cell transplantation. The 3-year DFS rate was 85.5 ± 4.2% **(A)**, and the 3-year OS rate was 86.8 ± 4.1% **(B)**.

During a median follow-up of 34.5 months (range 4–79 months), relapse occurred in eight patients. Relapses were temporally distributed: four within six months and four between 1 and 2 years post-transplantation. HR fusion genes were identified in six of the relapsed patients, comprising one case each of *KMT2A*::*AFDN*, *NUP98*::*KDM5A*, *NUP98*::*NSD1*, and *KAT6A*::*CREBBP*, plus two cases of *FUS*::*ERG*. Nine patients died during follow-up. The primary cause of death was disease relapse (n=7); the remaining two deaths were attributable to acute graft-versus-host disease (aGVHD) and sinusoidal obstruction syndrome (SOS), respectively. The estimated 3-year DFS and OS rates were 85.5% ± 4.2% and 86.8% ± 4.1%, respectively (**Figure 1**).

### Analysis of prognostic factors for survival

**Figure 2:**
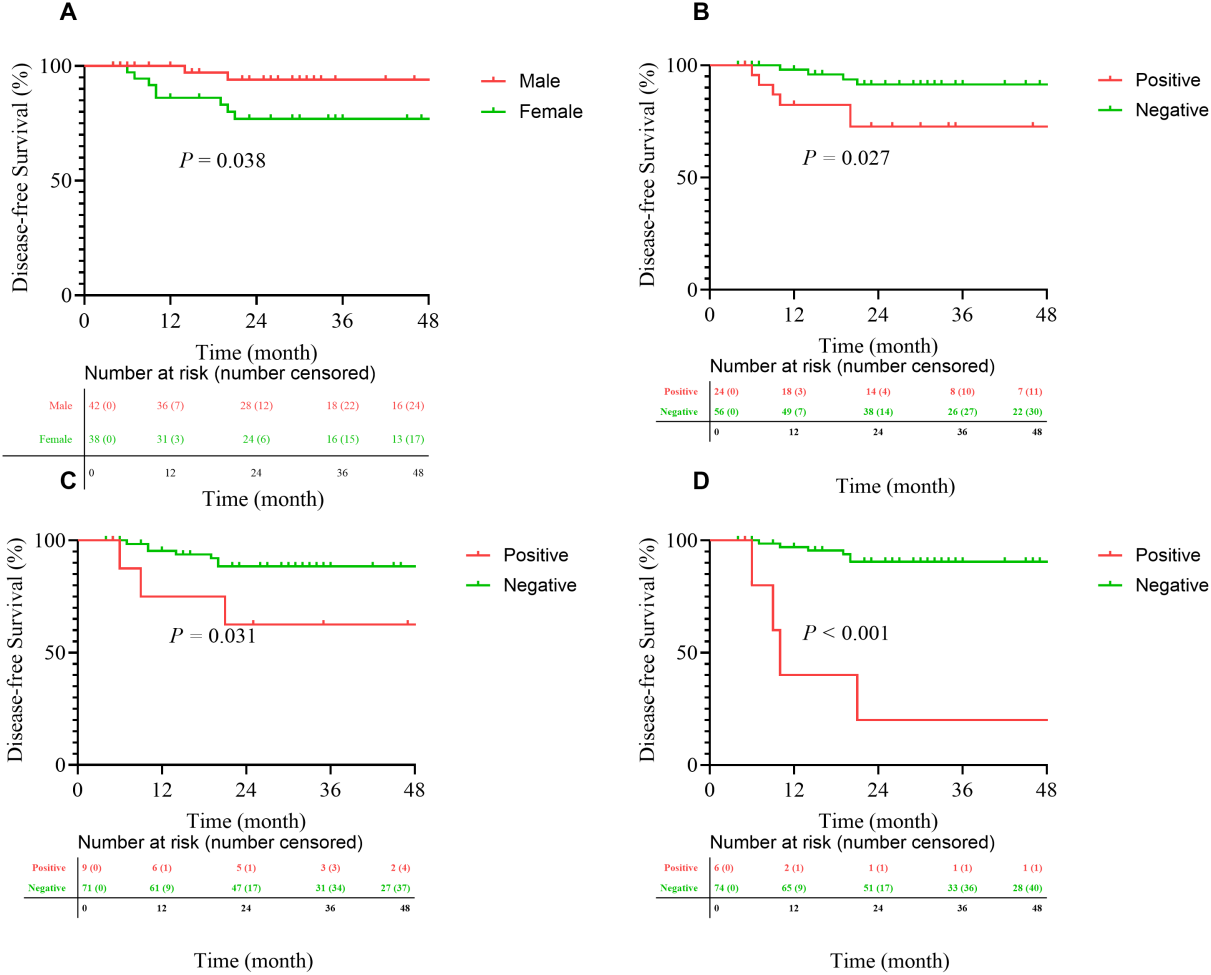
Survival curves of risk factors affecting 3-year DFS in pediatric AML patients. The 3-year DFS rates were 94.0% ± 4.1% for male patients and 77.0% ± 7.2% for female patients, showing a statistically significant difference (*P* < 0.05) **(A)**. The 3-year DFS rates were 72.7% ± 9.6% for patients positive for high-risk fusion genes and 91.4% ± 4.1% for those negative, with a significant difference (*P* < 0.05) **(B)**. For patients with MFC-MRD positive after the second induction chemotherapy, the 3-year DFS was 62.5% ± 17.1%, compared to 88.5% ± 4.1% for those with MFC-MRD negative, demonstrating a significant difference (*P* < 0.05) **(C)**. The 3-year DFS rates were 20.0% ± 17.9% for patients with pre-transplant MFC-MRD positive and 90.5% ± 3.7% for those with pre-transplant MFC-MRD negative, indicating a significant difference (*P* < 0.05) **(D)**.

**Figure 3:**
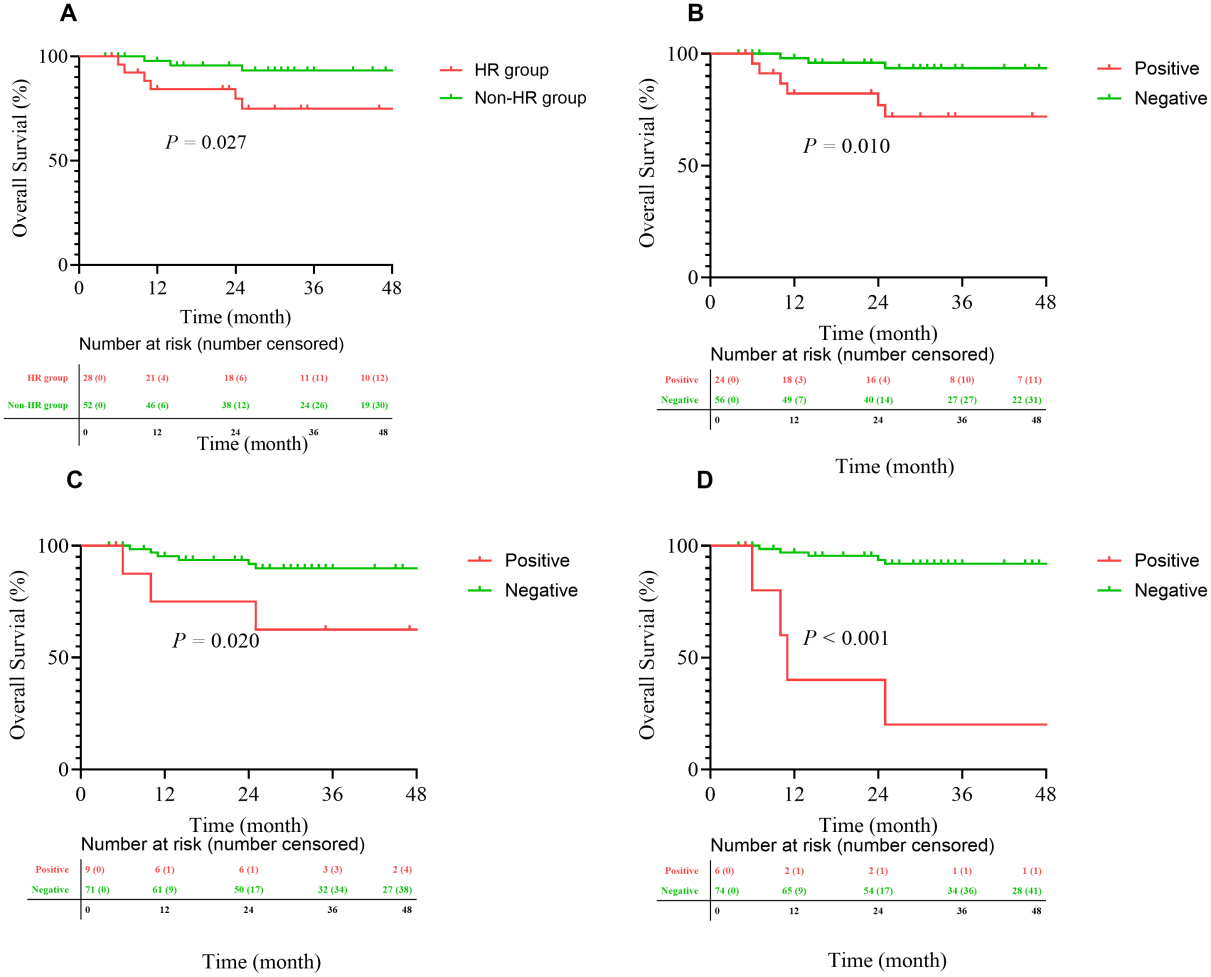
Survival curves of risk factors affecting 3-year overall survival (OS) in pediatric AML patients. The 3-year OS rates were 74.9% ± 9.0% for the high-risk group and 93.2% ± 3.8% for the non-high-risk group, demonstrating a statistically significant difference (*P* < 0.05) **(A)**. The 3-year OS rates were 71.9% ± 9.8% for patients positive for high-risk fusion genes and 93.6% ± 3.6% for those negative, with a significant difference (*P* < 0.05) **(B)**. For patients with MFC-MRD Positive after the second induction chemotherapy, the 3-year OS was 62.5% ± 17.1%, compared to 90.0% ± 3.9% for those with MFC-MRD negative, indicating a significant difference (*P* < 0.05) **(C)**. The 3-year OS rates were 20.0% ± 17.9% for patients with pre-transplant MFC-MRD positive and 92.0% ± 3.5% for those with pre-transplant MFC-MRD negative, showing a statistically significant difference (*P* < 0.05) **(D)**.

As summarized in **Table 1**, univariate analysis demonstrated that female sex, positivity for HR fusion genes, MFC-MRD positivity after the second induction, and pre-transplant MFC-MRD positivity were significant risk factors for inferior 3-year DFS (*P* < 0.05). Similarly, classification in the HR group, positivity for HR fusion genes, MFC-MRD positivity after the second induction, and pre-transplant MFC-MRD positivity were significant risk factors for worse OS (*P* < 0.05). Survival curves illustrating the impact of pre-transplant MFC-MRD status on 3-year DFS and OS are presented in **Figures 2** and **3**, respectively. Following univariate screening (*P* < 0.05), multivariate analysis confirmed pre-transplant MFC-MRD positivity as an independent adverse prognostic factor for both 3-year DFS and OS **Table 2**.

**Table 2:** Multivariable analysis of risk factors associated with 3-year DFS and OS in 80 patients.

Risk factor	DFS			OS		
	HR	95% CI	*P*	HR	95% CI	*P*
Sex	1.501	0.253­8.909	0.996			
High-risk fusion genes	4.077	0.853-19.485	0.078	3.826	0.217­67.574	0.360
MFC-MRD positive after cycle 2 induction	1.090	0.116­10.210	0.940	1.363	0.153­12.187	0.782
Pre-transplant MFC-MRD positive	14.304	1.892­108.155	0.010	15.847	2.036­123.328	0.008
Risk group				1.702	0.096­30.180	0.717

DFS, disease-free survival; OS, overall survival; HR, hazard ratio; MFC, multiparametric flow cytometry; MRD, measurable residual disease.

## Discussion

In this study, we conducted a retrospective analysis of 80 pediatric AML patients who underwent allo-HSCT during their first CR. With a median follow-up of 34.5 months, the 3-year DFS and OS rates were 85.5 ± 4.2% and 86.8 ± 4.1%, respectively. This indicates that overall allo-HSCT outcomes are encouraging when transplantation decisions are guided by risk stratification and early MFC-based MRD assessment. However, survival analysis further elucidated key prognostic factors. Multivariate analysis definitively established pre-transplant MFC-MRD positivity as an independent adverse prognostic factor for both 3-year DFS and OS. Furthermore, univariate analysis suggested that the presence of high-risk fusion genes and MFC-MRD positivity following the second induction chemotherapy cycle were also associated with inferior survival outcomes.

The utility of MFC-MRD monitoring in predicting prognosis in AML is well established (21). However, controversy remains regarding which treatment stage MFC-MRD positivity best serves as a prognostic factor. Rubnitz et al. reported that MFC-MRD positivity after the first induction chemotherapy is an independent adverse prognostic factor for both OS and event-free survival in pediatric patients (22). Other studies have also indicated that MFC-MRD positivity following the second induction chemotherapy is associated with poor survival outcomes (18, 23). In our study, through sequential monitoring of MFC-MRD at different chemotherapy stages and pre-transplantation, we demonstrated that pre-transplant MFC-MRD positivity serves as an independent adverse prognostic factor for both DFS and OS in children, which is consistent with findings reported by Bai et al (24). Therefore, our findings underscore the critical importance of maximizing tumor burden reduction prior to harnessing the GVL effect, in order to minimize the risk of post-transplant relapse. This conclusion does not negate the prognostic significance of MFC-MRD positivity after induction chemotherapy; rather, it contextualizes its value within a continuous, stage-specific therapeutic decision-making framework. The prognostic importance of post-induction MFC-MRD positivity lies in the early identification of patient cohorts who may require transplantation or in guiding initial risk stratification. In contrast, pre-transplant MFC-MRD positivity indicates a high risk of relapse following transplantation. Allo-HSCT can improve outcomes for patients with suboptimal early treatment responses. Therefore, the clinical implications of MFC-MRD positivity must be interpreted in conjunction with the specific treatment time point at which it is assessed (21).

Recent studies have demonstrated that monitoring the dynamic changes of MFC-MRD during the peri-HSCT period (before HSCT and approximately one month after HSCT) holds significant clinical value (25). patients who are MFC-MRD negative both before and after transplantation exhibit the lowest risk of relapse. In contrast, patients with persistent MFC-MRD positivity before and after transplantation, as well as those who convert from pre-HSCT negative to post-HSCT positive, have a poorer prognosis (25). Notably, patients who are MFC-MRD positive before HSCT remain at high risk of relapse even if they achieve early MRD clearance after transplantation (25). In this study cohort, the MFC-MRD conversion rate was 83% (5/6), aligning closely with previously reported data (26). Although subgroup analysis based on MFC-MRD status during the peri-HSCT period was not performed in our study, our findings indicate that pre-HSCT MFC-MRD positivity is an independent adverse prognostic factor affecting survival in pediatric patients. Therefore, for patients with pre-HSCT MFC-MRD positivity, preemptive therapeutic strategies are recommended irrespective of early post-HSCT MRD status.

Previous studies have indicated that FLAG-IDA induction chemotherapy is well-tolerated and effective in the treatment of both relapsed/refractory and newly diagnosed AML (27, 28). In the previously published multicenter C-HUNAN-AML-15 protocol, in which our institution participated, the CR rate after the first induction was 85.8%, and the MFC-MRD negativity rates were 62.6% after the first induction and 85.9% after the second induction (18, 29, 30). Since most patients in the prior study received FLAG-IDA induction, and FLAG-IDA showed significantly better survival outcomes compared with DAE induction (29, 30), the current cohort also primarily employed FLAG-IDA induction. In this cohort, the MFC-MRD negativity rates after the first and second induction chemotherapy were 62.5% and 88.7%, respectively, which are essentially consistent with the results of the multicenter study. These findings suggest that FLAG-IDA induction chemotherapy can achieve deeper responses and reduce the pre-transplant tumor burden, which is an important factor contributing to the favorable transplant outcomes observed here.

This study found that AML patients with HR fusion genes may still have a substantial risk of relapse after HSCT. In this cohort, the *KMT2A*-rearranged group (excluding *KMT2A*::*MLLT3*) achieved a 3-year OS of 93.8 ± 6.1%, indicating that HSCT can improve the prognosis of these patients. *NUP98* rearrangements account for approximately 7% of pediatric AML cases (31), which is comparable to the recently reported incidence of *NUP98*-rearrangements in adult AML patients in east asia (32). Pediatric patients with *NUP98*-rearranged AML generally have a poor prognosis, with a reported 5-year OS of only about 35% (31). Among the four patients with *NUP98*-rearrangements in this cohort, two relapsed within six months after transplantation, resulting in a post-transplant relapse rate of 50%. Additionally, both patients with *FUS*::*ERG* fusion gene in this cohort died due to relapse after transplantation. These observations are largely consistent with previous reports (31, 33–36). Given the very limited number of cases with these specific HR fusion genes (*NUP98* rearrangements, n = 4; *FUS*::*ERG*, n = 2; *KAT6A*::*CREBBP*, n = 1), these subgroup findings are exploratory and require validation in larger, preferably prospective, studies.

It should be emphasized that MFC-MRD and Mol-MRD each have their own advantages and disadvantages in the clinical application of AML; they are not substitutes but rather complement each other by leveraging their respective strengths. MFC-MRD is applicable to over 90% of AML patients, making it the most widely applicable, operationally simple and rapid, and low-cost MRD method (21). However, it has drawbacks such as antigen drift, inability to identify specific leukemic subsets, and complex analytical procedures (21). Mol-MRD offers high specificity, high sensitivity, relatively low cost, and ease of standardization, but it also has limitations: it is time-consuming, requires stable expression of target genes during treatment, is applicable to only a subset of patients due to RNA instability, and is suitable for only 30%–60% of the patient population (21). Therefore, HR fusion genes can be used to identify patients with high molecular biological risk, whereas pre-transplant MFC-MRD status directly measures the residual leukemic disease burden. Nevertheless, even among patients who achieve pre-transplant MFC-MRD negativity, those harboring HR fusion genes such as *NUP98* rearrangements or *FUS*::*ERG* still face a substantial risk of relapse, indicating that molecular risk stratification and MRD status provide distinct but complementary prognostic information.

This study has several limitations. First, it was a single-center, retrospective investigation with a relatively limited sample size (n = 80), which may have compromised the statistical power of certain subgroup analyses and restricted the generalizability of the findings. Notably, only 6 patients (7.5%) were pre-transplant MFC-MRD positive, and the number of events for specific HR fusion genes was extremely small. Second, the relatively wide confidence intervals observed in the multivariate Cox regression analysis (e.g., for DFS: HR = 14.304, 95%CI: 1.892–108.115; for OS: HR = 15.847, 95%CI: 2.036–123.328) indicate limited statistical precision and potential instability of the estimates, likely attributable to the small number of events. This further underscores the exploratory nature of our findings and the need for validation in larger cohorts. Third, the definition of *FLT3-ITD* positivity in this study (mutant-to-wild-type ratio ≥ 0.05) followed standard fragment analysis practice; however, cases with low-level *FLT3-ITD* (e.g., ratios between 1% and 5%) were classified as negative (20). The clinical and biological significance of such low-level mutations remains uncertain, and they may represent subclonal events with potential prognostic implications (20). This cut-off dependent classification should be considered a technical limitation. Fourth, the targeted RNA sequencing panel used for fusion gene detection does not provide full exon coverage for all relevant genes. Specifically, it does not capture the complete exon repertoire of genes such as *NUP98*, which may lead to under-detection of certain fusion variants involving non-covered exons.

In conclusion, pre-transplant MFC-MRD positivity is an independent adverse prognostic marker for survival in pediatric AML patients following allo-HSCT. Moreover, patients harboring HR fusion genes such as *NUP98*-rearrangements or *FUS*::*ERG* fusion genes may remain at risk of relapse even after successful transplantation, although this observation is based on a limited number of cases. These findings emphasize the importance of achieving deep remission to reduce tumor burden before transplantation and provide a rationale for intensified post-transplant management strategies, including maintenance therapy, for this HR population.

## Data Availability

The original contributions presented in the study are included in the article/**Supplementary Material**. Further inquiries can be directed to the corresponding author.
