# Supplementary material for: Pre-transplant measurable residual disease by flow cytometry is an independent prognostic factor in pediatric acute myeloid leukemia undergoing allogeneic hematopoietic stem cell transplantation
Source: Front Oncol. 2026 Jul 17;16:1864716. doi: 10.3389/fonc.2026.1864716 (PMC13423664; doi:10.3389/fonc.2026.1864716)
Supplement: Supplementary file 1 [file DataSheet1.docx]

**Additional file**


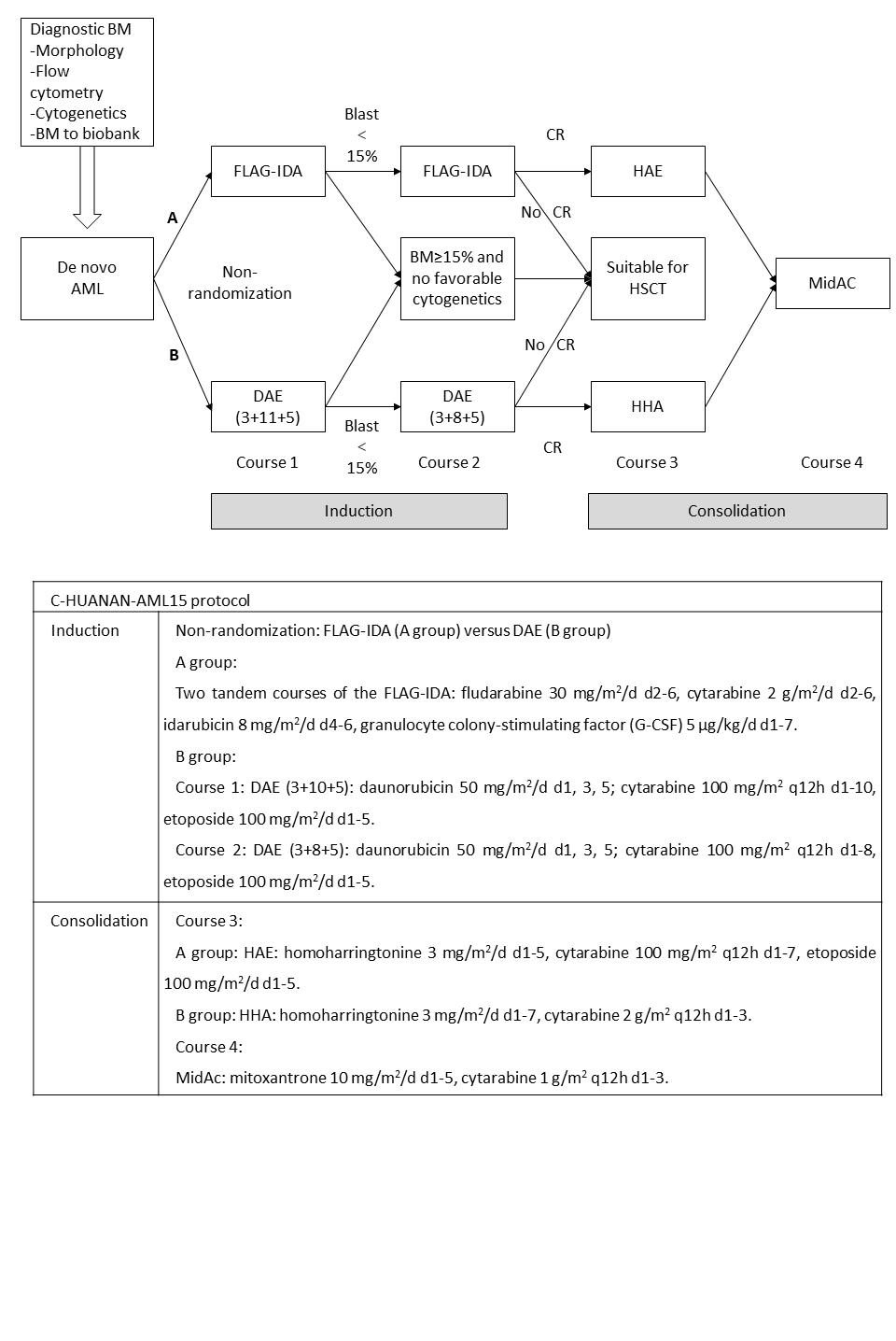


Supplementary Figure 1. Treatment schema for the C-HUANAN-AML 15 protocol. *Note: intermediate-risk patients with a sibling donor and high-risk patients were advised to undergo allo-HSCT; Children aged <1 year had all chemotherapy doses reduced by 25%. HSCT, hematopoietic stem cell transplantation.
