# Supplementary material for: Pre-transplant measurable residual disease by flow cytometry is an independent prognostic factor in pediatric acute myeloid leukemia undergoing allogeneic hematopoietic stem cell transplantation
Source: Front Oncol. 2026 Jul 17;16:1864716. doi: 10.3389/fonc.2026.1864716 (PMC13423664; doi:10.3389/fonc.2026.1864716)
Supplement: Supplementary file 2 [file Table1.doc]

| Component | HLA-Matched (Sibling/URD) | HLA-Haploidentical |
| --- | --- | --- |
| Conditioning (MAC) |  |  |
| Busulfan | Weight-based: <9 kg: 4 mg/kg/d; 9–16 kg: 4.8 mg/kg/d; 16–23 kg: 4.4 mg/kg/d; 23–34 kg: 3.8 mg/kg/d; >34 kg: 3.2 mg/kg/d; for 3 days (or 4 days if thiotepa omitted) | Same |
| Thiotepa | 10 mg/kg/d × 1 day | Same |
| Cyclophosphamide | 60 mg/kg/d × 2 days | 14.5 mg/kg/d × 2 days |
| Fludarabine | 30 mg/m²/d × 5 days | 40 mg/m²/d × 5 days |
| Semustine | 250 mg/m²/d × 1 day | Same |
| r-ATG | Total 5 mg/kg over 2–3 days | Same |
| GVHD Prophylaxis |  |  |
| Baseline | Cyclosporine + short-course MMF + MTX | Same |
| Additional | None | PT-Cy 50 mg/kg/d on days +3, +4 |

Supplementary Table S1.Conditioning regimen and GVHD Prophylaxis
