# Supplementary material for: Pre-transplant measurable residual disease by flow cytometry is an independent prognostic factor in pediatric acute myeloid leukemia undergoing allogeneic hematopoietic stem cell transplantation
Source: Front Oncol. 2026 Jul 17;16:1864716. doi: 10.3389/fonc.2026.1864716 (PMC13423664; doi:10.3389/fonc.2026.1864716)
Supplement: Supplementary file 3 [file Table2.doc]

| Characteristic | N (%) |
| --- | --- |
| Karyotype (n=80) |  |
| Normal | 18 (22.5%) |
| Abnormal | 62 (77.5%) |
| Abnormal karyotype distribution (n=62) |  |
| t(8;21) | 18 (29.0%) |
| 11q23/KMT2A rearrangements | 16 (25.8%) |
| inv(16)/t(16;16) | 7 (11.3%) |
| trisomy 8 (+8) | 6 (9.6%) |
| monosomy 7 or del(7q) (-7/7q-) | 4 (6.5%) |
| Complex karyotype | 4 (6.5%) |
| Other abnormalities | 7 (11.3%) |
| Gene fusions (n=80) |  |
| Any fusion detected | 59 (73.8%) |
| Fusion types among positive cases (n=59) |  |
| RUNX1::RUNX1T1 | 20 (33.9%) |
| CBFβ::MYH11 | 7 (11.9%) |
| KMT2A::MLLT3 | 7 (11.9%) |
| KMT2A::ELL | 5 (8.5%) |
| KMT2A::AFDN | 3 (5.1%) |
| KMT2A::MLLT10 | 3 (5.1%) |
| EVI1::MSD1 | 3 (5.1%) |
| Other fusions | 11 (18.6%) |
| High-risk fusion genes (n=80) |  |
| HR group by fusion | 28 (35.0%) |
| Gene mutations (n=80) |  |
| Any mutation detected | 66 (82.5%) |
| Mutation types among positive cases (n=66) |  |
| NRAS | 14 (21.2%) |
| KRAS | 9 (13.6%) |
| KIT | 7 (10.6%) |
| FLT3-ITD | 7 (10.6%) |
| WT1 | 6 (9.1%) |
| CEBPA (double mutation) | 6 (9.1%) |
| PTPN11 | 5 (7.6%) |
| ASXL1 | 5 (7.6%) |
| RUNX1 | 4 (6.1%) |
| TP53 | 3 (4.5%) |
| FLT3-TKD | 3 (4.5%) |
| Other mutations | 12 (18.2%) |

### Supplementary Table S2. Cytogenetic and molecular characteristics of the study cohort
